# *CD3G* or *CD3D* Knockdown in Mature, but Not Immature, T Lymphocytes Similarly Cripples the Human TCRαβ Complex

**DOI:** 10.3389/fcell.2021.608490

**Published:** 2021-06-25

**Authors:** Beatriz Garcillán, Patricia Fuentes, Ana V. Marin, Rebeca F. Megino, Daniel Chacon-Arguedas, Marina S. Mazariegos, Anaïs Jiménez-Reinoso, Miguel Muñoz-Ruiz, Raquel G. Laborda, Paula P. Cárdenas, Edgar Fernández-Malavé, Maria L. Toribio, José R. Regueiro

**Affiliations:** ^1^Department of Immunology, Ophthalmology and ENT, Complutense University School of Medicine and 12 de Octubre Health Research Institute (imas12), Madrid, Spain; ^2^Interaction with the Environment Program, Immune System Development and Function Unit, Centro de Biología Molecular Severo Ochoa, CSIC-UAM, Madrid, Spain

**Keywords:** *CD3G*, *CD3D*, shRNA knockdown, T-cell receptor (TCR), TCR assembly, T-cell progenitors, immunodeficiency

## Abstract

The human αβ T-cell receptor (TCR) is composed of a variable heterodimer (TCRαβ) and three invariant dimers (CD3γε, CD3δε, and ζζ/CD247_2_). The role of each invariant chain in the stepwise interactions among TCR chains along the assembly is still not fully understood. Despite the high sequence homology between CD3γ and CD3δ, the clinical consequences of the corresponding immunodeficiencies (ID) in humans are very different (mild and severe, respectively), and mouse models do not recapitulate findings in human ID. To try to understand such disparities, we stably knocked down (KD) *CD3D* or *CD3G* expression in the human Jurkat T-cell line and analyzed comparatively their impact on TCRαβ assembly, transport, and surface expression. The results indicated that TCR ensembles were less stable and CD3ε levels were lower when CD3γ, rather than CD3δ, was scarce. However, both defective TCR ensembles were strongly retained in the ER, lacked ζζ/CD247_2_, and barely reached the T-cell surface (<11% of normal controls) in any of the *CD3* KD cells. This is in sharp contrast to human CD3γ ID, whose mature T cells express higher levels of surface TCR (>30% vs. normal controls). *CD3* KD of human T-cell progenitors followed by mouse fetal thymus organ cultures showed high plasticity in emerging immature polyclonal T lymphocytes that allowed for the expression of significant TCR levels which may then signal for survival in CD3γ, but not in CD3δ deficiency, and explain the immunological and clinical disparities of such ID cases.

## Introduction

T lymphocytes detect the presence of antigens through a cell surface complex termed the T-cell receptor (TCR). This receptor is composed of a variable heterodimer (either TCRαβ or TCRγδ) responsible for ligand recognition and three invariant dimers (CD3γε, CD3δε, and ζζ/CD247_2_) that participate in the assembly and surface expression of the TCR complex and in the delivery of intracellular signals (Call et al., 2004). Binding of the peptide–MHC complex by the TCR results in CD3 conformational changes (Schamel et al., 2019) and the phosphorylation of tyrosine residues within the immunoreceptor tyrosine-based activation motifs (ITAM) of the cytoplasmic tails of the CD3 and ζζ/CD247_2_ chains (Love and Hayes, 2010), which eventually lead to the activation of several signaling cascades that result in T-cell activation and the induction of a cellular immune response (Brownlie and Zamoyska, 2013).

TCR assembly is an ordered process that begins in the endoplasmic reticulum (ER). CD3 heterodimers are formed through interactions between their extracellular Ig domains and associate with TCR chains through intramembrane contacts to form the hexameric TCR (αβγεδε), which is exported to the Golgi apparatus where it interacts with the ζζ/CD247_2_ homodimer (Dietrich et al., 1999; Wucherpfennig et al., 2010). The result is an octameric complex that is sorted to the plasma membrane. Correct folding and assembly is required for transport to the cell surface, and misfolded or single subunits and partial receptor complexes are retained in the ER and eventually retro-translocated to the cytosol for ER-associated degradation. Hexameric TCR ensembles that reach the Golgi apparatus but fail to assemble with ζζ/CD247_2_ homodimers are sorted to the lysosomal pathway for degradation (Bonifacino et al., 1990; Wileman et al., 1990, 1993; Letourneur and Klausner, 1992; Lee, 1998; Delgado and Alarcon, 2005).

CD3γ and CD3δ proteins are highly homologous, and in some vertebrates, they are a single CD3γ/δ chain (Göbel and Dangy, 2000). In humans, it has been shown that the intracellular and transmembrane, but not the extracellular, domain of CD3δ can substitute those of CD3γ for assembly and expression (Wegener et al., 1995). The functions of the individual CD3 chains for assembly and expression and the stepwise interactions among the TCR subunits are still not fully understood and differ between humans and mice.

Several studies have attempted to address these questions studying knockout (KO) mice [for CD3γ (Haks et al., 1998) or CD3δ (Dave et al., 1997)], immunodeficient patients (Perez-Aciego et al., 1991), mutant Jurkat T-cell lines (Geisler, 1992), or heterologous systems (Kappes and Tonegawa, 1991; Call et al., 2002; Szymczak et al., 2004), but they yielded contradictory results regarding the requirement of CD3γ and CD3δ proteins for surface TCR expression and signaling. Indeed, KO mice, mutant Jurkat T-cell lines, and reconstituted B-cell microsomes showed that TCR assembly, surface expression, and signaling required CD3γ and CD3δ proteins, whereas immunodeficient patients and reconstituted HeLa or 293T cells did not. Here, we knocked down (KD) CD3γ and CD3δ in mature and immature αβ T cells to analyze their role in TCR assembly and expression.

## Materials and Methods

### Cell Lines

*Mycoplasma*-free Jurkat E6-1 cells, a clone of the Jurkat JM CD4^+^ T-cell leukemia cell line established from the peripheral blood of a 14-year-old boy with acute T-cell leukemia (Schneider et al., 1977), was used because they express all TCR chains (Abraham and Weiss, 2004). The CH7C17 Jurkat cell line is a mutant of Jurkat E6-1 cell line, negative for TCR and reconstituted by transfection with a TCR specific for hemagglutinin peptide bearing Vα1.4 and Vβ3.1 TCR chains (Hewitt et al., 1992). The Herpesvirus Saimiri (HVS) CD3γ^–/–^ cell line was previously generated from peripheral blood lymphocytes (PBL) of a congenital CD3γ-deficient individual (Pacheco-Castro et al., 1998). Cells were grown in RPMI 1640 medium (HyClone, Logan, UT, United States) supplemented with 10% FCS and 2 mM L-glutamine (Life Technologies, Carlsbad, CA, United States); 400 μg/ml hygromycin B (Life Technologies) was supplemented for CH7C17 cell line, and 50 IU/ml human recombinant human IL-2 (provided by Craig W. Reynolds, Frederick Cancer Research and Development Center, National Cancer Institute, National Institutes of Health, Frederick, MD, United States) was supplemented every 2 days for the HVS CD3γ^–/–^ cell line. The HBP-ALL cell line was originally generated from a T-cell acute lymphoblastic leukemia patient. PBL were isolated from healthy donors with informed consent and IRB authorization. During the assay, all cells were maintained in RPMI 1640 medium supplemented with 10% FCS and 2 mM L-glutamine.

### Lentivirus Production, Transduction, and KD

To generate KD cells, the pLKO.1-CMV-TurboGFP^TM^ plasmid backbone containing a puromycin selectable marker, a turboGFP marker, and a short hairpin (sh) RNA specific for either CD3γ, CD3δ, or a nontarget (NT) control was purchased from Sigma-Aldrich (St. Louis, MO, United States) (Table 1). Lentiviruses were generated by co-transfecting HEK-293T cells (ATCC CRL-1573) with 6.6 μg pLKO.1 and the helper plasmids psPAX2 (4.8 μg Addgene # 12260, containing Gag and Pol) and pMD2.G (1.44 μg Addgene # 12259, containing VSV-G), kindly provided by Dr. Trono, using Lipofectamine LTX (Life Technologies) according to the manufacturer’s instructions. The growth medium was exchanged after 24h and the lentivirus-containing supernatant was harvested 24 and 48h later. The cell culture supernatant was used to infect Jurkat E6-1, CH717, or PBL cells in the presence of 8 μg/ml of polybrene (Sigma-Aldrich) or 2 μg/ml of polybrene for the HPB-ALL cell line. After 24 h, the viral particle-containing medium was removed and replaced with fresh medium. After 72h, 2 μg/ml puromycin (Sigma-Aldrich) was added to the Jurkat E6-1, CH717 and HPB-ALL (not for PBL) media for the selection of stable transfectants and tested regularly for KD status. To generate *CD3* KD early thymic progenitors (ETPs) for fetal thymic organ cultures (FTOC), shRNAs were cloned under the U6 promoter into pHRSIN lentiviral vector encoding GFP under the SFFV promoter (pHRSIN-SFFVp-GFP; Garaulet et al., 2013) and HEK-293 cells were co-transfected with the pHRSIN plasmid and the helper plasmids psPAX2 and pMD2.G indicated above. The supernatants were collected after 48h of transient transfection and ETPs were transduced on 30 μg/ml recombinant human (rh) fibronectin fragment CH-296 (RetroNectin) as described (García-Peydró et al., 2003), after overnight culture with rh interleukin 7 (rhIL-7; 100 IU/ml), rh stem cell factor (rhSCF; 100 IU/ml), and rh Flt3-ligand (rhFlt3-L; 50 IU/ml) (National Institute of Biological Standards and Controls, Herts, United Kingdom).

**TABLE 1:** shRNA sequences have been previously published in The RNAi Consortium (TRC) and were purchased from Sigma-Aldrich cloned into pLKO.1 vector.

shRNA	TRC number	Sequence (5′–3′)
shNT	Nontarget	CAACAAGATGAAGAGCACCAA
shCD3γ-1	TRCN0000057223	CGGCTTCCTAACTGAAGATAA
shCD3γ-2	TRCN0000057224	CTGGCTATCATTCTTCTTCAA
shCD3γ-3	TRCN0000057225	CAGAACTGCATTGAACTAAAT
shCD3γ-4	TRCN0000057226	CCACCTTCAAGGAAACCAGTT
shCD3γ-5	TRCN0000057227	GTATTACAGAATGTGTCAGAA
shCD3δ-1	TRCN0000057218	GCATCCATTGAGATGATAATA
shCD3δ-2	TRCN0000057219	CCGTGCAAGTTCATTATCGAA
shCD3δ-3	TRCN0000057220	GAGGACAGAGTGTTTGTGAAT
shCD3δ-4	TRCN0000057221	GTTGAGGAATGACCAGGTCTA
shCD3δ-5	TRCN0000057222	CACTGCTCTCAGACATTACAA

### Western Blotting and Immunoprecipitation

Cells were lysed in NP-40 lysis buffer (1% NP-40 from Sigma-Aldrich, 50 mM Tris–HCl pH 7.4, 100 mM NaCl, 10% glycerol, 2 mM MgCl_2_, 1 mM PMSF, 1 mM Na_3_VO_4_, 25 mM NaF, and 1 × Protease Inhibitor Cocktail Set I—Calbiochem from Sigma-Aldrich) or Brij96V lysis buffer (0.3% Brij96V from Sigma-Aldrich, 20 mM Tris–HCl pH 8, 150 mM NaCl, 10% glycerol, 4 mM EDTA, 1 mM PMSF, 2 mM Na_3_VO_4_, 10 mM NaF, and 1 × Protease Inhibitor Cocktail Set I—Calbiochem) for WB (Western blot) or IP (immunoprecipitation) experiments, respectively. For IP experiments, 2 mg lysates were incubated for 1 h at 4°C in the presence of 2 μg of antibody and protein-G magnetic beads (Dynabeads from Invitrogen, Carlsbad, CA, United States). Precipitates or 40 μg of total lysate were loaded in SDS-PAGE gels, transferred into PVDF membranes, and developed with antibodies indicated in each figure. For ER/Golgi transit experiments, half of the immunoprecipitated proteins were digested with recombinant Endoglycosidase H (EndoH) from *Streptomyces plicatus* (Sigma-Aldrich), which cleaves within the chitobiose core of high mannose and some hybrid oligosaccharides from N-linked glycoproteins. To this end, samples were incubated with 10 mU EndoH for 3 h at 37°C, resuspended in nonreducing loading buffer, and analyzed by WB.

Antibodies against alpha tubulin (Sigma), CD3γ (C20 from Santa Cruz Biotechnology, Dallas, TX, United States, EPR4517 from Abcam, Cambridge, MA, United States, and HMT3.2, kindly donated by Dr. R.T. Kubo, La Jolla Institute for Allergy and Immunology, CA, United States), CD3δ (M20 from Santa Cruz, APA 1/2 kindly provided by Dr. B. Alarcón, Centro de Biología Molecular Severo Ochoa, Madrid, Spain, and EP4426 from Abcam), CD3ε (EPR5361(2) from Abcam), or ζζ/CD247_2_ (448, kindly provided by B. Alarcon, Centro de Biología Molecular Severo Ochoa, Madrid, Spain) were used. Blots were visualized using Odyssey Infrared Imaging System (LI-COR Biosciences, Lincoln, NE, United States). Images of IRDye680 and IRDye800 fluorescence were obtained using the 700- and 800-nm channels. The quantification of signal intensity was carried out with Image Studio software.

### Flow Cytometry

Multiparametric flow cytometry was performed with standard procedures. For extracellular flow cytometry, monoclonal antibodies against CD3 (clone HIT3A APC and SK7 PE from Becton Dickinson, Franklin Lakes, NJ, United States), CD3ε (UCHT-1 PE-Cy5 from Beckman Coulter, Brea, CA, United States; Hit3A-APC from BioLegend, San Diego, CA, United States), TCRαβ (BMA031 APC from Miltenyi, Auburn, CA, United States), TCR γδ (clone 5A6.E9 Pe-Cy5 from Life Technologies), Vβ3 (CH92 FITC from Beckman Coulter), Vβ8 (56C5 PE from Beckman Coulter), Cβ1 (JOVI-1 sc-53196 from Santa Cruz Biotechnology), CD2 (S5.2 APC from BD), and HLA-class I (W6/32 PE-Cy7 from eBioscience, San Diego, CA, United States) were used. For intracellular staining, cells were fixed with 2% PFA for 30 min at 4°C, permeabilized with 0.2% saponin for 15 min at 20–22°C and stained with anti-CD3γ or anti-CD3δ (clones EPR4517 or EP4426 from Abcam) followed by a secondary fluorescent antibody (anti-rabbit PE from Beckman Coulter). Cells were stained with one single antibody per tube, labeled cells were acquired on FACSCalibur flow cytometer (Becton Dickinson) and the data analyzed with FlowJo software (TreeStar, Ashland, OR, United States).

### Fluorescence Microscopy

Cells were seeded in circular cover glasses, preincubated with 0.005% poly-L-lysine (Sigma-Aldrich) for 30 min at 37°C, and rinsed several times. Cells were then fixed with 4% p-formaldehyde in PBS for 5 min and, after rinsing, permeabilized with 0.1% Triton X-100 (Sigma-Aldrich) in PBS (both steps at room temperature, 20–22°C). Nonspecific binding sites were blocked by incubation for 1 h in PBS containing 10 μg/ml human IgG and a commercial blocking reagent (Roche, Basel, Switzerland). Incubation with primary TCRβ antibody (JOVI-1) or secondary anti-mouse antibody AF594 (Invitrogen) was done for 1 h or 30 min at 20–22°C, respectively. Appropriate isotype-matched IgG or normal serum was used in parallel as the control. Stained cells were mounted in Vectashield^®^ -DAPI solution (Vector Laboratories, Burlingame, CA, United States) and visualized under a Leica SP-2 AOBS confocal microscope (HCX PL APO × 63 1.4 oil-immersion objective; 1.400,000, numerical aperture) and TCSNTV software (Leica Microsystems, Wetzlar, Germany). Images were software processed with ImageJ.

### Statistical Analysis

Statistical analysis, including regression comparisons, was performed using GraphPad software. All quantitative data are expressed as the mean ± standard deviation (SD). Statistical differences between groups were compared using one-sample Student’s *t*-test. *P* < 0.05 (^∗^) was considered to indicate a statistically significant result. ^∗∗∗^*P* < 0.01.

### Isolation of Human Thymocytes and Hybrid Human–Mouse Fetal Thymic Organ Cultures

Experiments were performed in accordance with the procedures approved by the Spanish Research Council Bioethics Committee. Normal human postnatal thymocytes were isolated from thymus fragments removed during corrective cardiac surgery of patients aged 1 month to 4 years, after informed consent in accordance with the Declaration of Helsinki. Thymocyte suspensions were subjected to density centrifugation using Ficoll-Hypaque (Lymphoprep, Axis-Shield PoC AS), and Early T-cell progenitors (ETPs) were then isolated by immunomagnetic cell sorting using the Dynal CD34 Progenitor Cell Selection System (Life Technologies). Fetal thymic organ culture (FTOC) assays were performed as described (García-Peydró et al., 2003). Briefly, thymic lobes from 14.5-day-old Swiss mouse embryos were seeded (one lobe/well) in Terasaki plates (Nunc, Roskilde, Denmark), treated with deoxyguanosine (d-Guo, Sigma), and co-cultured with shNT- or shCD3δ-transduced human ETPs (1–2 × 10^4^ cells/lobe). After 48 h of culture in hanging drops (day 0 of FTOC), lobes were cultured on gelfoam/filters for up to 5 weeks. T-cell generation was analyzed by flow cytometry on electronically gated GFP^+^ cells recovered from different lobes pooled at indicated time points.

## Results

### Generation and Characterization of *CD3G* or *CD3D* KD T-Cell Lines

**FIGURE 1:**
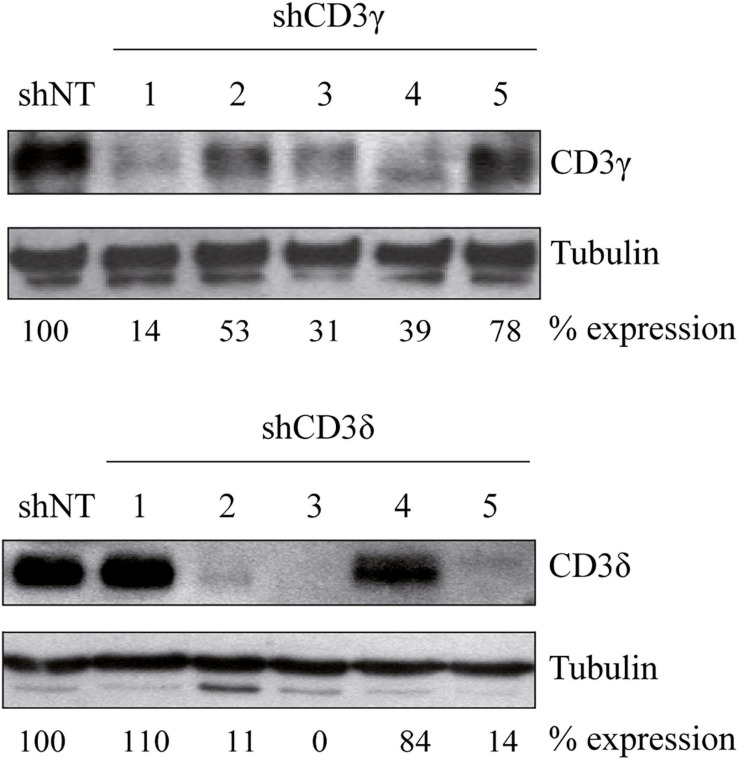
Generation of *CD3G* or *CD3D* KD T-cell lines. Jurkat E6-1 cells were transduced with five different shRNA for CD3γ or CD3δ and one non-target shRNA (shNT). Cells were lysed in NP-40 buffer after 7 days in culture and 40 μg of total protein was loaded. Membranes were incubated with antibodies against alpha tubulin (Sigma), CD3γ (Santa Cruz Biotechnology), or CD3δ (Santa Cruz Biotechnology). Numbers below each panel indicate the percentage of each chain expression relative to shNT normalized to the loading control (tubulin).

To investigate the importance of CD3γ and CD3δ in TCR assembly and expression, we generated *CD3G* or *CD3D* KD T-cell lines using five different shRNA sequences for inhibition of *CD3* expression (Table 1). The Jurkat E6-1 T-cell line was used to screen the different shRNAs. To this end, cells were transduced with lentiviral vectors carrying shRNA specific for CD3γ (shCD3γ), CD3δ (shCD3δ), or a nontarget shRNA (shNT). After at least 7 days in culture, cells were lysed in the presence of the selective agent puromycin and analyzed by WB for the expression of the targeted CD3 chain. Band intensity analysis showed that different sequences led to different degrees of KD (Figure 1). For further analysis, we selected lines shCD3γ-1 and shCD3δ-3, for the most efficient inhibition relative to shNT.

**FIGURE 2:**
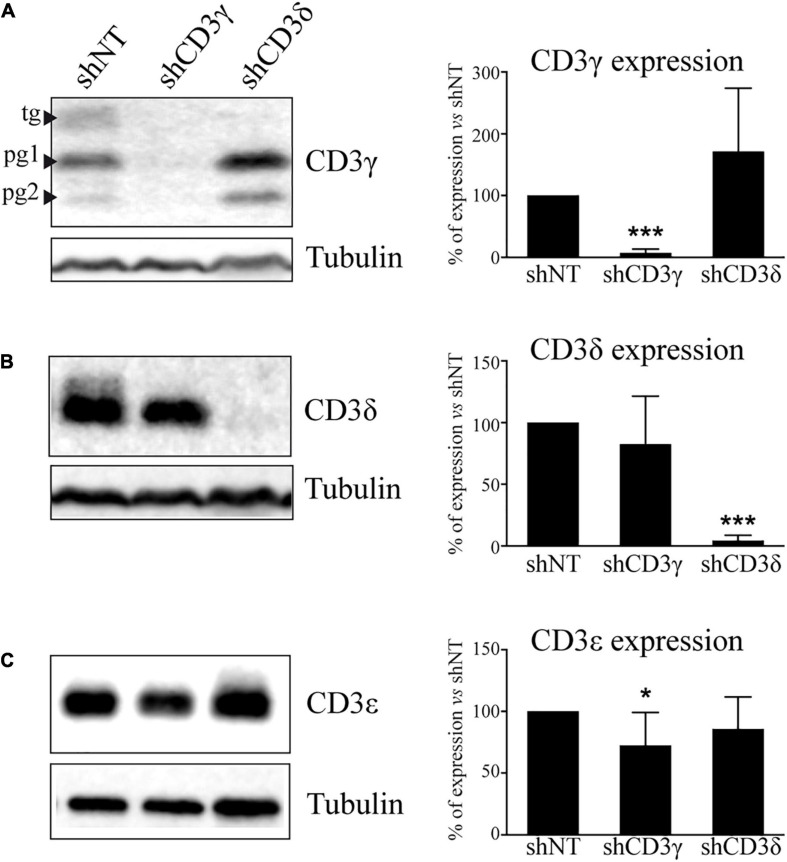
CD3 protein expression in *CD3G* or *CD3D* KD T-cell lines. Jurkat E6-1 cells were transfected with shCD3γ, shCD3δ, and shNT. Cells were lysed in NP-40 buffer after several days in culture with puromycin and 40 μg of total protein was loaded. Membranes were incubated with antibodies against tubulin (Sigma) and **(A)** CD3γ (Abcam), **(B)** CD3δ (Santa Cruz Biotechnology), or **(C)** CD3ε (Abcam). Band analysis and numbers as in Figure 1. Left: a representative experiment. Right: quantification of at least 10 different experiments; data are mean values + SD. Tg, totally glycosylated; pg, partially glycosylated. Note: all glycosylated forms of CD3γ were quantified together.

To fully characterize the impact of the KD on the TCR, we analyzed in stable KD T-cell lines the level of expression of all CD3 chains by WB. The results showed a marked selective impairment in the expression of CD3γ or CD3δ proteins in Jurkat cells treated with shCD3γ or shCD3δ, respectively, as compared with shNT (Figures 2A,B). The analysis of CD3ε expression in KD lines showed a slight but significant 30% reduction of CD3ε in the *CD3G*, but not in the *CD3D*, KD cell line (Figure 2C).

### TCR Assembly in *CD3G* or *CD3D* KD T-Cell Lines

**FIGURE 3:**
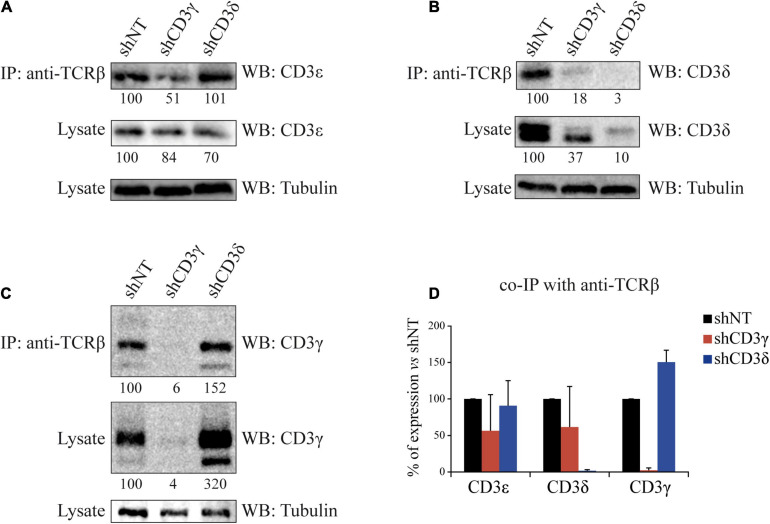
Co-IP of TCRβ and CD3 chains in *CD3G* or *CD3D* KD T-cell lines. Cells were lysed in Brij96V buffer, and 3 mg of total protein was immunoprecipitated (IP) with the anti-TCRβ mAb JOVI-1; the immunoprecipitates and whole lysates were resolved in 15% SDS-PAGE gels, transferred into PVDF membranes, and immunoblotted with antibodies against alpha tubulin and CD3ε in **(A)**, CD3δ in **(B)**, or CD3γ in **(C)**. Analysis and numbers as in Figure 1. **(D)** Quantification of three different experiments; data are mean values + SD.

To evaluate the impact of the KD of CD3γ or CD3δ chains on TCR assembly, we analyzed TCR stoichiometry by co-immunoprecipitation (co-IP) with anti-TCRβ and WB with CD3 chain-specific antibodies. Compared with shNT cells, when *CD3G* was KD only 51% of CD3ε and 18% of CD3δ co-IPed with TCRβ (Figures 3A,B, top lanes). However, when *CD3D* was KD, similar amounts of CD3ε (101%) and higher amounts of CD3γ (152%) were incorporated (Figures 3A,C top lanes). Thus, higher amounts of γε dimers were incorporated to (α)β after *CD3D* KD than were δε dimers after *CD3G* KD (summarized in Figure 3D). We conclude that TCR ensembles are more stable when CD3δ, rather than CD3γ, is scarce.

### TCR ER/Golgi Transit in *CD3G* or *CD3D* KD T-Cell Lines

**FIGURE 4:**
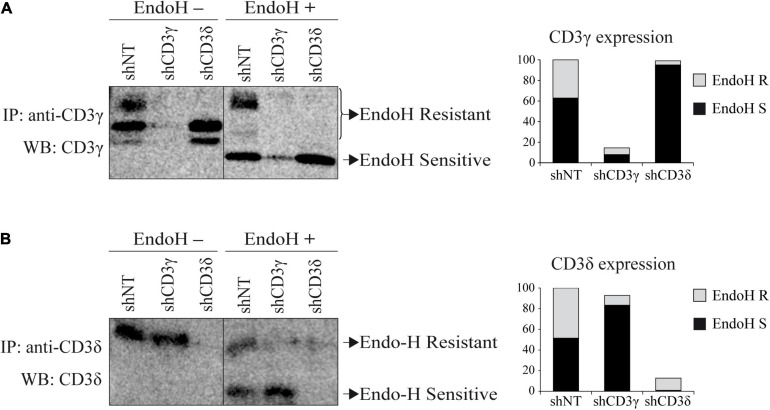
EndoH sensitivity of CD3 chains in *CD3G* or *CD3D* KD T-cell lines. **(A)** Cells were lysed in Brij96V buffer and immunoprecipitated with anti-CD3γ (HMT3.2). Half of the immunoprecipitated proteins were treated with EndoH. Samples were analyzed in 15% SDS-PAGE gels and immunoblotted with anti-CD3γ antibody (Abcam). The graph represents the quantification of EndoH-resistant (R, Golgi-associated) and EndoH-sensitive (S, ER-associated) species. **(B)** Cells were immunoprecipitated with anti-CD3δ (APA1/2) antibody and immunoblotted with anti-CD3δ (Abcam). Sample preparation, treatment with EndoH, and analysis as in **(A)**.

To determine the subcellular compartment location of the remaining CD3 chains after the KD, we studied TCR ER/Golgi transit by analyzing CD3 chain sensitivity to EndoH after IP and WB with CD3 chain-specific antibodies, because the Golgi, but not the ER, protects CD3 oligosaccharide side chains from EndoH digestion. Both forms of CD3γ and CD3δ, post-ER (EndoH resistant) and ER resident (EndoH sensitive), can be observed in shNT cells (Figure 4). In contrast, *CD3D* KD essentially blocked CD3γ in the ER (only 3% exited the ER, Figure 4A). Similarly, in *CD3G* KD cells, only 10% of CD3δ was able to reach the Golgi (Figure 4B). Our results indicate that both CD3γ and CD3δ chains in both KD cell lines were EndoH sensitive and, therefore, ER resident, suggesting that TCR complexes in the absence of CD3γ or CD3δ are retained in the ER.

### ζζ/CD247_2_ Incorporation to the TCR in *CD3G* or *CD3D* KD T-Cell Lines

**FIGURE 5:**
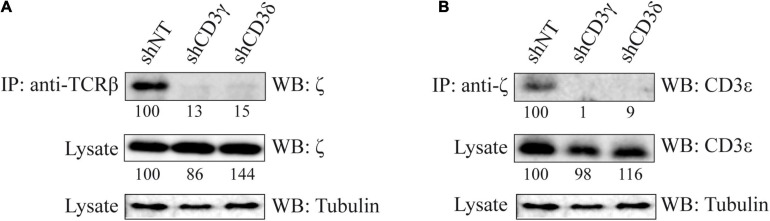
Co-IP of ζζ/CD247_2_ and other TCR chains in *CD3G* or *CD3D* KD T-cell lines. **(A)** Cells were lysed in Brij96V buffer, 3 mg of total protein was immunoprecipitated with anti-TCRβ mAb (JOVI-1), and the immunoprecipitates and whole lysates were resolved in 15% SDS-PAGE gels and immunoblotted with antibodies against ζζ/CD247_2_ (448) or alpha tubulin (Sigma). **(B)** Immunoprecipitation was done with anti-ζ and immunoblot with mAb against CD3ε (Abcam) or α-tubulin (Sigma). Band analysis and numbers as in Figure 1. The tubulin loading control is shared in **(A,B)** for illustrative purposes.

To study the last step in TCR assembly, namely, ζζ/CD247_2_ incorporation, its association to TCRβ and CD3ε was analyzed by IP and WB. The results showed that the clear co-IP of TCRβ and CD247 (Figure 5A) and of CD247 and CD3ε (Figure 5B) observed in shNT cells was absent in both *CD3G* and *CD3D* KD T-cell lines. We conclude that TCR complexes in both *CD3* KD cells do not incorporate ζζ/CD247_2_ homodimers.

### Surface Expression of the TCR Complex in *CD3G* or *CD3D* KD T-Cell Lines

**FIGURE 6:**
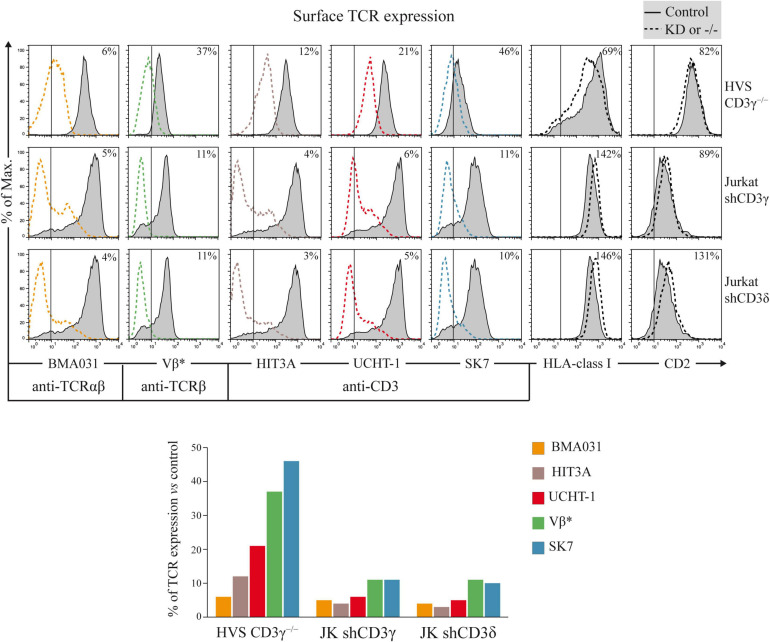
Surface TCR expression in *CD3G* or *CD3D* KD T-cell lines. Top: cells were stained with labeled TCR- or CD3-specific mAb (BMA031, HIT3A, UCHT-1, Vβ*, and SK7) or with TCR-independent control mAb (anti-HLA-class I and anti-CD2). Expression was analyzed in HVS-CD3γ^–/–^, Jurkat shCD3γ, and Jurkat shCD3δ cells (dashed lines) compared with their relevant controls (HVS-CD3γ^+/+^ or Jurkat shNT, gray histograms, the latter shown twice as it is the control cell line for both *CD3G* and *CD3D* KD cell lines). The vertical line in each panel indicates the upper limit of background fluorescence using isotype-matched irrelevant mAb. The numbers in each histogram indicate geoMFI ratios (×100) relative to controls. Bottom: bar representation of TCR/CD3 geoMFI ratios relative to controls. *Vβ3- or Vβ8-specific mAb was used to stain the TCRβ chain in HVS or Jurkat cells, respectively, because they have different *TCRB* rearrangements.

**FIGURE 7:**
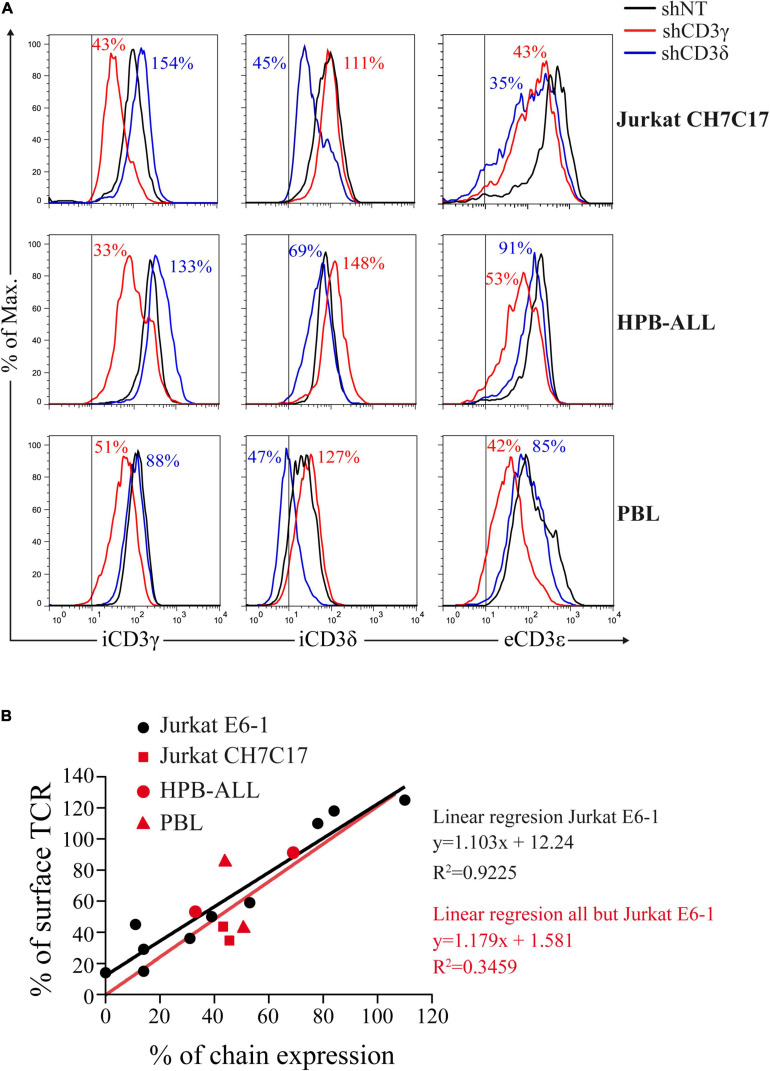
*CD3* KD and surface TCR expression correlate in T cells other than Jurkat E6-1. **(A)** Comparative intracellular (i) CD3γ or CD3δ expression and extracellular (e) CD3ε expression in the indicated T cells transduced with shCD3γ, shCD3δ, or shNT. Vertical lines indicate the upper limit staining of the isotype control. Numbers indicate % expression vs. control (shNT). **(B)** Correlation between CD3γ or CD3δ chain expression and surface TCR, expressed as % of expression vs. shNT. Data in **(A)** (red symbols) in comparison with Jurkat E6-1 data (black dots) and comparative linear regression. The slopes are not significantly different (*P* = 0.9046).

**FIGURE 8:**
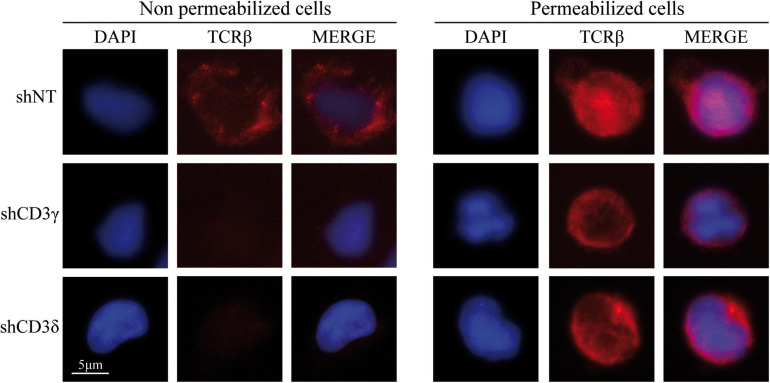
Extracellular (left) and intracellular (right) TCRβ expression in *CD3G* or *CD3D* KD T-cell lines. Jurkat E6-1 shNT, shCD3γ, and shCD3δ cells were left untreated (unpermeabilized) or were treated with Triton X-100 (permeabilized) and incubated with anti-TCRβ antibody (JOVI-1) and anti-mouse AF594 secondary antibody before analysis by fluorescence microscopy. Nuclei were stained with DAPI.

To assess the impact of *CD3G* or *CD3D* KD on surface TCR expression, flow cytometry was performed using a broad panel of mAb against the TCR or CD3 chains in the Jurkat E6-1 KD and in a HVS CD3γ^–/–^ cell line. The results indicated that the KD cell lines with reduced intracellular amounts of either CD3γ or CD3δ, expressed decreased surface TCR levels detected with several antibodies, with between 5 and 10% of surface TCR relative to controls in both cell lines (Figure 6). These observations demonstrated a critical requirement of CD3γ and CD3δ for surface TCR expression in the Jurkat E6-1 T-cell line. The effects of *CD3* KD on surface TCR levels were also analyzed in primary T cells and T-cell lines other than Jurkat E6-1 (Jurkat CH7C17 and HPB-ALL), and the results were comparable to Jurkat E6-1 T cells (Figure 7), further supporting that CD3γ and CD3δ are required for normal surface TCR expression in mature T cells. These results were confirmed by confocal microscopy for TCRβ, which was detectable both intracellularly and extracellularly in Jurkat E6-1 shNT cells, but only intracellularly in *CD3G* and *CD3D* KD cell lines (Figure 8). The shNT unpermeabilized cells stained for TCRβ showed clear evidence of patching, likely as a result of crosslinking with the secondary antibody (see Figure 8 legend). In contrast, TCRβ was absent on the cell surface of both CD3 KD cells. Surprisingly, surface TCR was less affected in the HVS CD3γ^–/–^ cell line than in KD lines (Figure 6). We conclude that TCR complexes do not reach the surface in any of the *CD3* KD cells. Therefore, the stark differences in disease presentation in patients lacking CD3γ (mild) or CD3δ (severe) cannot be ascribed to differences in the role of each chain in mature T cells. Rather, there must be critical signaling or expression differences whereby CD3γ, but not CD3δ, can be replaced early in T-cell development. Indeed, as explained below, CD3δ, but not CD3γ deficiency, showed a complete block in the differentiation of T cells in the patient’s thymus (Dadi et al., 2003).

### Surface Expression of the TCR Complex in *CD3D* KD Early T-Cell Progenitors

**FIGURE 9:**
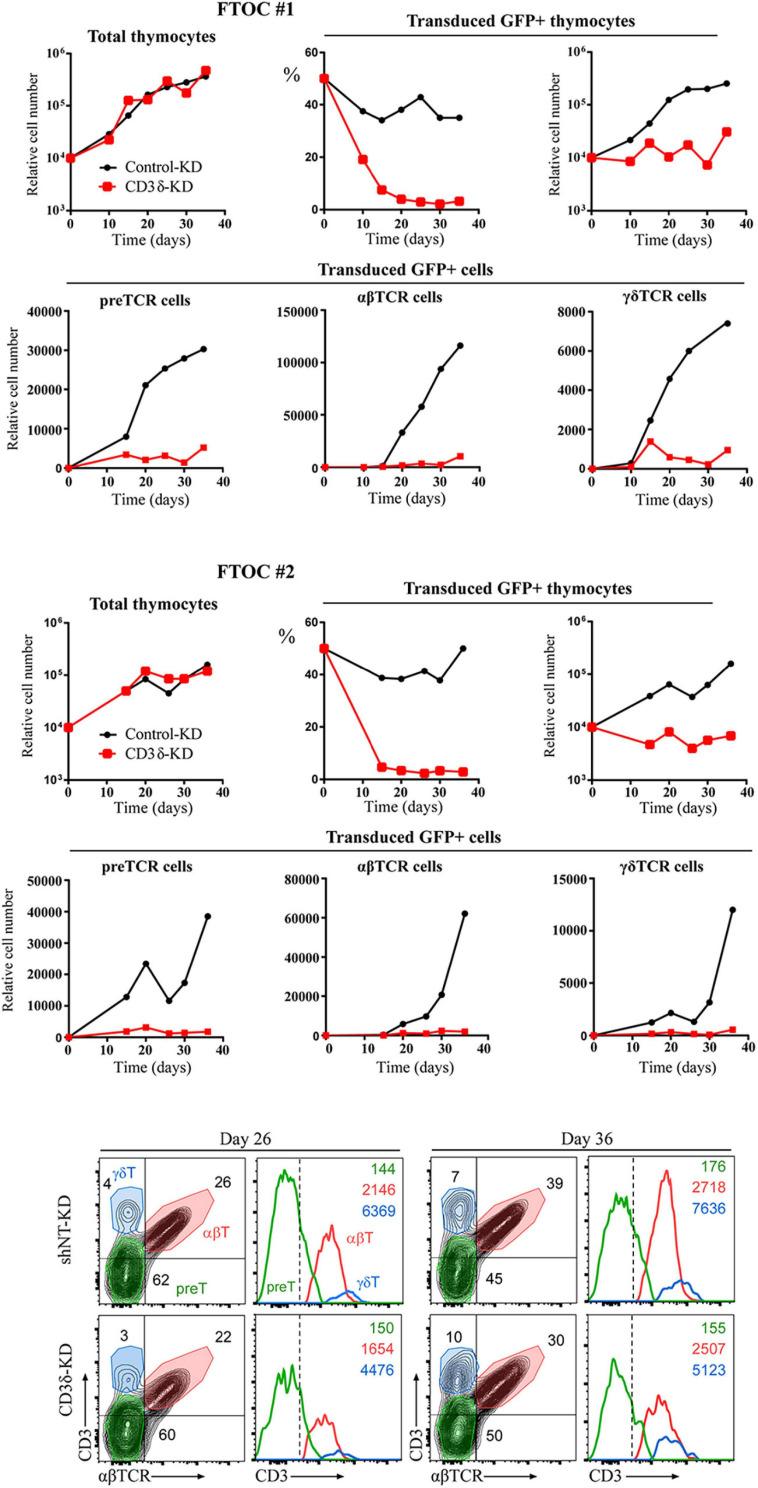
Fetal thymus organ cultures (FTOC) of shCD3δ-3 vs. shNT-transduced human T-cell progenitors. Top and middle: mouse embryo thymic lobes were seeded with shCD3δ-3 or shNT-transduced CD34^*hi*^ early T-cell progenitors from human thymus and cultured for up to 5 weeks. T-cell generation was analyzed on electronically gated GFP^+^ transduced cells recovered from different lobes pooled at the indicated time points in two independent experiments. Bottom: representative (FTOC 2) flow cytometry analysis of CD3 vs. αβTCR expression on electronically gated GFP^+^ cell progenies at the indicated days. Numbers in biparametric histograms indicate percentages of CD3^+^ αβTCR^+^ (αβ T cells), CD3^–^/low αβTCR^–^ (including pre-TCR^+^ pre-T cells) and CD3^+^ αβTCR^–^ (γδ T cells). Percentages of γδ T cells were independently confirmed by CD3 vs. γδTCR expression analysis. Numbers in monoparametric histograms indicate MFI values for each indicated subset.

Our results in human mature T cells showed that both CD3γ and CD3δ are required for surface TCR expression. Our co-IP experiments showed that nascent TCRs begin to form but are unable to incorporate ζζ/CD247 dimers and are retained in the ER. These results do not recapitulate the corresponding congenital CD3 deficiencies in terms of surface TCR expression (>30% vs. normal controls). We thus hypothesized that intrathymic plasticity of emerging polyclonal lymphocytes in patients might allow some TCR expression in a proportion of T cells, which could then signal for survival in CD3γ, but not in CD3δ deficiency. To directly address this hypothesis, we transduced early T-cell progenitors from human thymus with shCD3δ-3 or shNT before FTOC. We chose this KD strategy in order to assess both T-cell development and TCR expression in a single experiment, since shCD3γ would be expected to be milder in both aspects. The results indicated that *CD3D* KD strongly impaired human T-cell development in FTOC (Figure 9, top and middle), as observed also in human congenital CD3δ deficiencies. However, the few T cells (pre-T, αβ, or γδ) that did emerge from the organ cultures expressed comparable, albeit lower (around 80% vs. shNT or control KD), TCR ensemble levels. These results support the hypothesis of intrathymic TCR expression plasticity of emerging polyclonal T lymphocytes, which can then signal for survival in CD3γ, but not in CD3δ deficiency, and explain the immunological and clinical disparities of these ID cases.

## Discussion

In contrast with all other reported CD3 ID due to complete protein deficiencies, most CD3γ-deficient individuals can live well into their 30s without hematopoietic stem cell replacement (Marin et al., 2015). In these individuals, late diagnosis, mild T lymphopenia, low but significant TCR expression, and autoimmune features are common. These limited immunological consequences are at odds with what is observed when the highly homologous CD3δ chain is absent: early diagnosis, severe T lymphopenia, no TCR expression, and urgent transplantation. This suggests that there must be strong biochemical or functional differences between them. Conversely, mouse KO models show that CD3γ, but not CD3δ, is critical for early thymic development, and therefore, do not recapitulate human findings. We reasoned that by KD of each chain in human mature T cells we could find relevant differences. However, we found that both *CD3G* and *CD3D* KD T-cell lines were unable to incorporate ζζ/CD247_2_ to their TCR, and thus, showed a comparable decrease in surface TCR expression. We also observed in the analysis of ER/Golgi transit that *CD3G* and *CD3D* KD completely blocked TCR ensembles since CD3δ and CD3γ, respectively, did not reach the Golgi. Therefore, the immunological and clinical disparities of ID cases may be associated with biochemical or signaling differences between CD3γ- and CD3δ-deficient TCR ensembles in immature rather than mature T lymphocytes, for instance, at the pre-TCR level. To support this hypothesis, analysis of gene expression in thymocytes isolated from one patient diagnosed with SCID caused by CD3δ deficiency showed that a small number of gene products known to regulate T-cell development were substantially altered in the patient, as compared with the control. Of particular interest, patient’s thymocytes contained twice as much precursor T-cell receptor a (pTa) gene transcript as did control thymocytes. Since the pTa gene is expressed exclusively by immature thymocytes, these results indicate a block early in the differentiation of T cells in the patient’s thymus. Such a block could cause immature CD4^–^CD8^–^ double-negative thymocytes to accumulate (Dadi et al., 2003).

The analysis of CD3ε expression in KD lines showed a slight but significant reduction of CD3ε in the *CD3G*, but not in the *CD3D*, KD cell line (Figure 2) and less stable (α)βδε than (α)βγε TCR ensembles (Figure 3). A potential explanation for both events may be the faster degradation of (α)βδε TCR compared with (α)βγε TCR. However, such differences did not have an impact on later assembly events or surface TCR expression, which were similar in both KD cell lines.

TCR ensembles barely reached the T-cell surface in any of the *CD3* KD Jurkat cells (<11% of normal controls). A mutagenized Jurkat T-cell line lacking CD3γ called JGN (for Jurkat Gamma Negative; Geisler, 1992) also lacked surface TCR expression, supporting our findings. However, surface TCR was much less affected in the CD3γ ID-derived HVS T-cell line than in our KD lines or in JGN (>30% vs. normal controls), and this is the case too for the scarce peripheral mature T cells in partial CD3δ ID (Gil et al., 2011; Garcillán et al., 2014). Why does natural CD3γ deficiency differ from Jurkat CD3γ KD? Our hypothesis is that mature T cells in CD3γ deficiency are the result of selection from a very diverse and polyclonal repertoire of immature T cells with differently rearranged variable TCR chains, so that those expressing sufficient surface TCR can be selected to reach the peripheral blood compartment, albeit self-tolerance may be affected (Rowe et al., 2018). By contrast, Jurkat is a clone, and thus, there is no diversity to choose from when KD is performed, which would explain the crippling effect of CD3γ KD on TCR expression. Other cell lines and primary T cells showed similar results (Figure 7). Primary T cells, although polyclonal, do not have TCR-dependent selection mechanisms as immature T cells do. To test our hypothesis in polyclonal cells, FTOC *CD3D* KD experiments were performed, in which the few T cells (pre-T, αβ, or γδ) that did emerge from the cultures expressed comparable TCR ensemble levels (Figure 9). These results support the hypothesis of intrathymic TCR expression plasticity of emerging polyclonal T lymphocytes, which can then signal for survival in CD3γ, but not in CD3δ deficiency and explain the immunological and clinical disparities of these ID cases.

In conclusion, our results show that both CD3γ and CD3δ are required for surface TCR expression in human mature T cells. Our co-IP experiments show that nascent TCRs begin to form but are unable to incorporate ζζ/CD247_2_ dimers and are retained in the ER. In contrast, the FTOC experiments show that the lack of those chains early in development, acting on emerging polyclonal repertoires, allows for some TCR expression plasticity and further T-cell development when signaling from incomplete TCRs is feasible, as is the case in CD3γ, but not in CD3δ, ID.

Our findings could give rise to potential future therapeutic advances, including the transient reduction of CD3δ or CD3γ expression by KD of mature T cells as a novel immunosuppressive therapy, particularly in those diseases with overactive T cells, such as autoimmune or alloimmune disorders.

## Data Availability Statement

The original contributions presented in the study are included in the article/supplementary material, further inquiries can be directed to the corresponding author/s.

## Ethics Statement

The studies involving human participants were reviewed and approved by Comité Ético de Investigación Clínica del Hospital Universitario 12 de Octubre de Madrid. Written informed consent to participate in this study was provided by the participants’ legal guardian/next of kin.

## Author Contributions

BG, PF, MT, and JR conceived the experimental study. BG designed and performed most of the experiments, and analyzed the data. PF did the FTOC studies (Figure 9) under the supervision of MT. RM, DC-A, and RL did TCR comparative experiments in cell lines and fresh human T cells (Figure 7) under the supervision of PC. MM, MM-R, and AJ-R contributed with substantial technical help to several figures. EF-M and AM contributed substantially to the manuscript revision and figures’ elaboration. JR and BG wrote the manuscript. All authors participated in the manuscript revision before submission.

## Conflict of Interest

The authors declare that the research was conducted in the absence of any commercial or financial relationships that could be construed as a potential conflict of interest.
